# The challenge of compiling data profiles to stimulate local preventive health action: a European case study from child safety

**DOI:** 10.1007/s00038-015-0665-z

**Published:** 2015-03-05

**Authors:** Denise Alexander, Michael Rigby, Mika Gissler, Lennart Köhler, Morag MacKay

**Affiliations:** 1 Department of International Health, School for Public Health and Primary Care (CAPHRI), Maastricht University, Maastricht, The Netherlands; 2 Nordic School of Public Health, Gothenburg, Sweden; 3 National Institute for Health and Welfare, Helsinki, Finland; 4 European Child Safety Alliance, Birmingham, UK

**Keywords:** Child health, Injury, Prevention, Surveillance

## Abstract

**Objectives:**

Positive recent experience of presenting comparative child safety data at national level has instigated policy action in Europe. It was hoped a Child Safety Index could quantify how safe a community, region or locality is for its children in comparison with similar areas within Europe, as a focus for local targeted action.

**Methods:**

Validated indicators proposed by previous European projects identified from areas of child injury prevention, such as road safety, burns or poisoning, were selected to give a balanced profile, and populated from available published data. An index using a sub-score for each specific injury topic was proposed. The indicators’ presentation, sensitivity and appropriateness were considered, as well as data availability.

**Results:**

Satisfactory indicators were not identified for all areas and very few local area data were available. This forced the researchers to conclude that at present, constructing a reliable Child Safety Index for use at the local level is not feasible.

**Conclusions:**

There is a worrying lack of data available at the sub-national level to support injury prevention, evaluate interventions, and enable informed local decision making.

## Introduction

Unintentional injury is one of the most important public health issues for children and young people. It is the largest cause of death for children over 5 years of age, and a major cause of disability, pain, and stress to children and their families (Sethi et al. 2008). Despite the recognised importance of this issue, we know comparatively little about what it is in regions and communities that makes children more vulnerable to unintentional injury, or how policies can be prioritised based on evidence of need.

There are still major challenges in measuring and identifying the true extent of injury prevalence to children in Europe. At national level, the European Child Safety Alliance has achieved successes in producing and promoting comparative national data analyses (MacKay and Vincenten 2009, 2012), but at more local level there are few data available. To address this, the creation of a sub-national Child Safety Index was proposed as part of a European Commission (EC) project entitled Tools to Address Childhood Trauma, Injury and Children’s Safety (TACTICS) (European Child Safety Alliance 2014). The aim of the Child Safety Index was to help regions and communities evaluate injury risk and the safety of children and young people, by providing an input to facilitate decision making on positive actions.

### Under-valued importance of local data

Effective preventive public health relies upon good national and international data (The Prevention Institute 2008). However, whilst national data give essential information about the wider picture of a country’s health status, they have limitations. National data alone cannot identify specific populations or geographical locations in real need of action by identifying areas of deprivation or high prevalence of injury occurrence. National actions to reduce injury are important, but policy prioritisation and other action at more local level have a significant part to play. One means of determining this is to identify and accurately measure the injury prevalence to children in Europe on a sub-national scale (Tamburlini et al. 2002). Work by Safe Kids Worldwide (2014) has identified the important contribution of local community and individual effort towards making communities safer for young people. This is echoed by the European Healthy Cities Network (de Leeuw et al. 2014), a central goal of which is to strengthen caring a supportive environments through local community responsibility; and by the current World Health Organisation Health 2020 strategy (2013), which emphases community resilience and empowerment. An important means of doing this is to make available information on a community level, allowing relevant decisions to improve health, and reduce inequalities in local settings. Alongside this is the central aim of the TACTICS project, which is to find a means of democratising data, making information available and understandable to public and professionals equally and accessibly. This fits with a long-standing recognition of the importance of community empowerment in promoting health (Laverack and Labonte 2000). Thus, the intended universality of the Child Safety Index was important, its indicators and data should be equally available across and between countries, using validated components and existing available data.

### Child safety index concept

The Child Safety Index was to be compiled of relevant existing validated indicators of child safety and injury, but using these indicators at a sub-national level. This would allow a degree of comparability between the international, national and local situations that could be useful in identifying highest need, promoting solutions and evaluating interventions. The Index would also be built using existing routine data, available uniformly across a country and the continent. There has been much recent work into harnessing and measuring data on a local level. The European Urban Health Indicators System Part 2 (EURO-URHIS-2) project made an important contribution to the field of local health indicators; and is similar to TACTICS in that it seeks to validate the indicators by means of using existing population-based registries and databases. However, this project differs from TACTICS in its use of a combination of routine and survey-based work (EURO-URHIS-2 2009) and its primarily urban focus. The Child Safety Index aimed to measure small units of a whole country, not just the urban elements of a country, and to be affordable and accessible using only existing data. Other locally focussed initiatives rely upon survey data (Pettman et al. 2014; Stöcklin et al. 2013), but these data often cannot be reliably generalised or there is no commitment to regular data collection so trends over time cannot be visualised. Systematic review and meta data analysis play a vital role in our knowledge of sub-national regions, particularly in subjects that are difficult to measure, such as child maltreatment (Barth et al. 2013). These means of data gathering can be costly and impractical, particularly if a repeatable and regular data analysis is required to demonstrate a trend. Data retrieved in this way are not often readily accessible to a lay audience. Using hospital episode data can also be problematic, despite its use in a number of other public health analyses (Palacio-Viera et al. 2013), but the techniques are not easily transferrable to the specific subject of injury, due to the high number of injuries not presented at hospital (Peden et al. 2008) and because of the small numbers involved on a local level. Thus, there is a potential need for an injury-focused index of child safety.

It is known that much of the action required to tackle injury must be undertaken locally, even where policy is made at the national level (Tamburlini et al. 2002; Sethi et al. 2010a). Using only national data would, therefore, be insufficient to describe specific local risks and inform meaningful targeted action. The hope for the Child Safety Index was that it would provide an immediate comparative quantification of an area’s child safety merits and disadvantages, and thus indicate priorities for action. However, the attempt to create this Child Safety Index illuminated fundamental gaps in the data about children and young people as well as about injuries, problems of meaningful small area analysis, and difficulties in finding a practical and ‘real life’ solution to a well-researched problem.

## Methods

### Definition of the child population

For the purposes of this exercise, the definition of a child as a person up to the age of 18 years, as stated by the United Nations Convention on the Rights of the Child (Office of the High Commissioner for Human Rights 1989), was used. Alongside this definition, the TACTICS team recognised that the Child Safety Index must be flexible enough to take into account the vast differences in needs, abilities and exposures encompassed by this age group. In addition, when defining and choosing indicators to include in the Child Safety Index, account was taken of the influences of the family and other wider environmental influences in children, such as the physical and school environments (Glasgow Centre for Population Health 2013).

### Identifying potential indicators

The first stage was to identify potential indicators that could be used to form the Child Safety Index. To do this, we conducted a literature search of PubMed using the initial search strategy of ‘safety’ AND ‘community’ AND ‘local’ AND ‘health’, which retrieved 147 abstracts. In addition to this, we conducted specific searches to find evidence relating specific injury types, as detailed by the Child Safety Report Card work carried out by the European Child Safety Alliance (MacKay and Vincenten 2012). These were: ‘poisoning’ AND ‘child’ AND ‘local’ (61 abstracts); ‘falls’ AND ‘child’ AND ‘local’ (44 abstracts); ‘water safety’ AND ‘child’ AND ‘local’ (4 abstracts); (‘moped’ OR ‘scooter’) AND ‘child’ AND ‘local’ (4 abstracts); ‘transport’ AND ‘child’ AND ‘local’ (14 abstracts). Papers were searched worldwide from the past 10 years. We also conducted searches of relevant literature to identify indicators from the Child Friendly Cities initiative (UNICEF 2014), the Child Health Indicators for Life and Development (CHILD) project (Rigby and Köhler 2002) and other European Union (EU) initiatives, including the EU-funded Child Safety Action Plan (MacKay and Vincenten 2007, 2010) and its Child Safety Report Card indicators (MacKay and Vincenten 2012), the Environmental Health Information System (ENHIS) (World Health Organisation Regional Office for Europe 2014), the Child Environmental and Health Action Plan for Europe (CEHAPE) (World Health Organisation Regional Office for Europe 2004), the Adolescence and Risk Taking (AdRISK) project (EuroSafe 2014), Children’s health and environment: a review of the evidence (Tamburlini et al. 2002), the European Report on Preventing Violence and Knife Crime Among Young People (Sethi et al. 2010b), the Health Evidence Network (HEN) (Health Evidence Network 2004), and Public Health Action for a Safer Europe (PHASE) (2008). These, together with other indicators listed on the Research Inventory of Child Health in Europe (RICHE) project (2014), were examined and all those relating to injury or safety were identified. All of the above projects contained indicators pertinent to children and to safety against injury, and all were based on scientific rationale and had defined data constructs and potential sources.

### Policy and outcome indicators

Two types of indicators were identified, policy and statistical indicators. These are not mutually exclusive; they provide different, but valuable, types of information. Policy indicators are powerful at national level, showing for instance the existence of specific safety legislation or regulation. However, they can be more problematic at local level, as either the national law applies uniformly or, for devolved legislation, they require collation of municipal and local laws. There are also important issues surrounding the enforcement of laws and local laws, particularly as research suggests that enforcement of such laws can differ between localities (Erickson et al. 2014). Statistical indicators, showing the outcome, or mechanism of injury in a particular area can provide extremely rewarding data. However, these data are not routinely collected on a sub-national scale. In terms of statistical indicators, mortality data were excluded from the Child Safety Index project because of the very small numbers, which would lead to issues concerning statistical reliability and confidentiality risks including circumstantial identification.

### Sub-national indicator compilation

In selecting the national indicators to be used on a sub-national scale, we drew upon experience from the Nordic School of Public Health in Gothenburg, Sweden, in re-analysing national level indicators to a municipal population level (Köhler 2006, 2012c) and by the National Institute of Child Health in Hungary at a regional level (Pall 2004). Köhler (2006, 2012c) used national indicators developed by the CHILD project (Rigby and Köhler 2002) in a new way, to map children’s health and wellbeing in small geographical areas, such as municipalities and even sub-municipalities. This model has been successfully used in practice (Köhler 2012b, 2013; Köhler and Henriksson 2013). Pall (2004) used the CHILD indicators at a regional level with some success, identifying areas of elevated risk that were not known when using national data alone (Pall 2004). The resulting long list of potential indicators was then categorised in terms of type of injury they describe. The categories included several from the Child Safety Report Cards (European Child Safety Alliance 2014) such as drowning and water safety; road safety; burns and scalds; falls; poisoning; choking, suffocation and strangulation—as well as others viewed as important (such as products and safety in the home; and alcohol, self-harm and violence).

### Testing of candidate indicators

A scheme was devised to ‘test’ each indicator to assess its suitability to be included, so that indicators could be chosen consistently and with a degree of scientific rigour. The selection criteria consisted of four dimensions: representation, data and baseline availability, statistical meaning, and utility. In terms of representation, it was felt essential that indicators should be capable of representing their category in a systematic way within an integrated index. Data need to be available for a reasonable number of localities at the level selected, and statistically valid within the size/time interval/frequency of available data, even if techniques such as moving averages had to be applied. The reliability of the indicator definition and accuracy of compilation are also statistical factors that were taken into account. Finally, in terms of utility, each indicator in the index should be valuable in describing and measuring what is child injury risk or protection, and to conform to a number of criteria, outlined in Table 1.

**Table 1:** Criteria agreed upon by the TACTICS project to establish utility levels of each proposed indicator for inclusion in the Child Safety Index (Milan, Italy 2012)

Utility criteria
1. In use and with a rationale
2. Significant trauma, or outcome burden to individual child
3. Significant burden to family and society
4. Risk occurs in normal life, not specialist activities
5. Regularity and repeatability to enable trend analysis
6. Topic amenable to effective action
7. Understandable to individuals and community
8. Understandable to policy makers and politicians

These selection criteria made up a scoring system that was applied to each identified indicator in a methodology similar to that which had successfully been used in the CHILD project (Rigby and Köhler 2002). The indicators on the long list were discussed by the TACTICS partners and scored with a point if they met the terms of the representation, data and baseline availability, and statistical meaning requirements, and additionally with a point for each of the elements that make up the utility requirement.

### Creation of the index

The intention of the TACTICS project was to draw into one analytic tool the safety-related proposals scattered through a number of recent proposals, into a single composite index, which would incorporate all dimensions of injury risk. The team identified and reviewed already-existing composite health indexes to establish whether their methodology was suitable for adoption to produce a child injury risk index to aid prevention measures. An evaluation of existing composite indexes was carried out, and the construct of a number of respected indexes in European child health was examined, including An Index of Child Wellbeing in Europe (Bradshaw and Richardson 2009) and Comparing Child Wellbeing in OECD Countries (Bradshaw et al. 2006). Discussions were held to consider issues such as weighting of items, and the use of a framework model.

Once a well-conceptualised and operationalised indicator was identified it was evaluated in terms of its standardisation and interrelationships between other measures and representativeness of the issue. After careful consideration, there was no hierarchy placed on the indicators chosen, and therefore no weighting. This lack of weighting was because very few indicators have the evidence to assign justifiable higher value to them; and because the potential elements of the Index change in their respective relevance to injury risk or prevention in terms of age, geography and a young person’s immediate social and cultural environment (Köhler 2012a). Constructing the index in this way meant that for each domain the indicators would be combined into a single summary figure, effectively a sub-index; and the sub-indexes would then be combined into a single summary index that is understandable and simple to interpret.

## Results

### Number of initial indicators

After interrogating the projects listed above, 106 potentially relevant indicators were identified. They were selected if they corresponded to children and to injury risk factors, using the agreed-upon Child Safety Index categories. A degree of pragmatism was used to ensure as wide a spread of indicators as possible at this stage.

The majority of indicators found were policy indicators as opposed to indicators of exposure or safety measures. In some categories, there were few or even no statistical, population-based indicators, as illustrated in Table 2.

**Table 2:** Potentially relevant indicators from European project sources (2002–2008), by Child Safety Index category and indicator type (discussed in TACTICS meeting Milan, Italy, 2012)

Child safety index category	Total indicators in feeder projects	Of which	
		Policy indicators	Statistical indicators
Alcohol, self-harm and violence	5	–	5
Bullying and violence	10	1	9
Burns and scalds	8	6	2
Choking, suffocation and strangulation	6	6	–
Drowning	9	9	–
Falls	6	6	–
Poisoning	10	5	5
Products and safety in the home	12	12	–
Road safety	36	26	10
Workplace injury	4	–	4
Total	106	71	35

Table 2 illustrates the challenging findings that appeared from the outset. Some areas were very strongly represented, but other topics were very poorly covered. Moreover, the statistical indictors were patchy and poorly distributed. For example, road safety is over-represented, whilst key areas such as drowning and falls had no proposed measures. Moreover, there were no indicators describing crime, perceptions of safety, playground injuries or sport-related injuries despite their importance to communities and to children’s safety. Whilst over-representation can be handled by selection of items, gaps cannot be remedied that way. Added to this, many of the policy indicators related to presence or absence of national policies such as legislation, or tax incentives, so there is little scope for local versions of these.

### Number after suitability tests

The chosen indicators were then analysed in terms of the devised scoring system, concerning representation, local area data availability, statistical validity and utility. Of these indicators, not one scored highly enough on all of the criteria to be suitable for the creation of a Child Safety Index.

In terms of representation, most of the indicators conformed to this criteria, because they were already in use and tested as indicators as part of other European or Global projects. However, because the Child Safety Index is concerned with sub-national data, it was felt that a number of indicators, which purely describe national policy or laws would not be applicable to inclusion in the index. Once these indicators were deleted from the list, 54 indicators remained.

In terms of local area availability, there were considerable obstacles encountered. It was important to define the statistical units to determine the scale of sub-national data that could be used by the Index. In Europe, the statistical units commonly used are the Nomenclature of Territorial Units for Statistics (NUTS) (Eurostat 2012). The project team attempted to use NUTS units on as small a level as possible to test the remaining 54 indicators, but this proved problematic. Even the higher level NUTS units were challenging to use in countries with smaller populations, for example Ireland or Finland. In these countries, NUTS units were primarily geographical clusterings of smaller, lower-level units and bore little relationship to local administrative boundaries, or to other data sources such as health data, which made it difficult to generate real meaning from the information gained. Using smaller, lower-level NUTS units was problematic because data are too sparse for statistics to be reliable, and issues of confidentiality become pertinent, as well as fewer of the data sources being published at this level. Thus, it proved highly problematic to identify a NUTS level small enough to be meaningful in terms of local relevance, and large enough to be commonly defined and statistically robust.

The chosen indicators were already tested on a national scale and were robust in terms of their statistical meaning. They were also concise in terms of the utility scoring system devised by the project. However, the statistical meaning for many indicators became compromised when used sub-nationally, due to lack of data or extremely small numbers. In most countries, many issues relating to child safety, such as playgrounds, parks, fencing, traffic calming, and school crossing patrols, are influenced by decisions and investment at a very local level such as municipality or county. Clearly, the kind of statistical indicators being considered would not be meaningful at this level, whilst larger areas of several million overall population would be statistically more robust, but would be remote from local decision making.

## Discussion

The results suggested that the creation of meaningful sub-national public health analyses to support effective local action, in this case a Child Safety Index, is generally problematic in Europe, except in the largest federal countries where, at best, regional data in units as large as many EU Member States seem valid. This study showed that the objective of using existing indicators and populating the Index with existing data was not feasible on a meaningful sub-national level in Europe. This was disappointing and in some respects, surprising. The obstacles encountered in the work on the Child Safety Index, however, become interesting areas of discussion. Not least because they highlight the urgent need for sub-national data on injury to children to be collected and analysed.

### Indicators that describe injury

Injury has a multitude of risk factors, determinants and behavioural influences, and its complexity requires a range of indicators to adequately describe it. The indicators identified by the project were fragmented across many initiatives in Europe, which impacts upon their influence on injury levels and may account for the imbalance of coverage in the Child Safety Index. There was a strong emphasis on road safety, but very few indicators to describe poisoning, burns or injuries due to leisure activities. In addition to this, the choice of indicators can itself be complex. Adding in specific indicators—describing the presence or absence of a safety strategy, such as “the presence or absence of walk-to-school initiatives”, may prevent the inclusion of other local preventive initiatives. However, using a broad indicator such as “Policies to ensure safe transportation to school” may not yield useful data to allow communities to take effective action. Specific indicators often do not have universal definitions or have different contexts, meaning that a ‘walk-to-school’ scheme in one locality is very different to one in another locality thus rendering comparison difficult. Some important environmental determinants of injury and safety were missing entirely—such as perception of crime and perceived neighbourhood safety (World Health Organisation 2014). What is needed are indicators of exposure to injury on a sub-national scale, the creation of which is a task that was well outside the original EU project or any other attempt to utilise already available and published data from official sources.

### Analysis of injury data

Exposure data and incidence data for injury are not straightforward to interpret. For example a higher than average number of cycling injuries may mean dangerous roads; or that there is an extremely active and inclusive cycling culture in the area, with a large number of children enjoying the physical, social and mental benefits of regular exercise using their bicycles. Finally, in terms of an index, combining these extremely disparate elements would run a real risk of compromising its value. Given the complexity of causality, the combination of such heterogeneous elements such as drowning incidence and a lack of infant car restraint law would arguably be too artificial a construct to have any meaningful or major influence on policy or action, other than possibly highlighting the need for investigative action where overall rates of injury are high.

The shortcomings of available indicators to comprise an index mean that any resulting data would not be interpretable in a meaningful manner. The limited data available on a sub-national level only exacerbates the difficulty. A large geographical area is likely to be diverse in its character, containing rural and urban areas, and areas of differing economic prosperity. With available data, it is not possible to identify communities at real need of specific intervention.

Injury prevention needs to take place at a community level alongside national level policy changes to improve safety (Tamburlini et al. 2002; Sethi et al. 2010a). But the data available were not helpful for local decision-making purposes. In addition, injury risk has been shown to be highly influenced by socio-economic status and by the environment in which a child lives, plays or goes to school (Sethi et al. 2010a). The data available at present are not able to measure or describe social inequalities in risk or inequalities in exposure to injury that research has demonstrated exist (Laflamme 2012). Data that are only available at a higher level cannot effectively describe the extent of community cohesion. Communities themselves are not homogenous. A geographical index would mask these effects, but stratifying any index would cause problems with small numbers and data reliability.

### Conclusions

This study shows that production of meaningful local public health data, particularly child safety and injury data, provides a conundrum. Only local data are strongly relevant for influencing appropriate local actions, but availability of such data in meaningful form is shown to be limited, and what is present is not comparable with that of other local areas. There have been a number of published research studies that measure child health topics; but these are predominantly survey based; are limited to certain locations only, or use and provide data that are challenging for a variety of interested parties to interpret. The results of these studies align with our findings that although there have been successes in measuring specific child health topics, the nature and limitations of these studies though intrinsically successful, provide no added insights into how to make common measures related to safety available to stakeholders in an accessible way from routine and publicly available data. In addition, facts such as degree of exposure to specific hazards or preventive measures, or local demographic variants, can easily be masked. This continues to be a subject needing further research.
